# EVI1 modulates oncogenic role of GPC1 in pancreatic carcinogenesis

**DOI:** 10.18632/oncotarget.20601

**Published:** 2017-09-01

**Authors:** Mariko Tanaka, Shumpei Ishikawa, Tetsuo Ushiku, Teppei Morikawa, Takayuki Isagawa, Makoto Yamagishi, Hiroyuki Yamamoto, Hiroto Katoh, Kimiko Takeshita, Junichi Arita, Yoshihiro Sakamoto, Kiyoshi Hasegawa, Norihiro Kokudo, Masashi Fukayama

**Affiliations:** ^1^ Department of Pathology, Graduate School of Medicine, The University of Tokyo, Tokyo, Japan; ^2^ Department of Genomic Pathology, Medical Research Institute, Tokyo Medical and Dental University, Tokyo, Japan; ^3^ Department of Cardiovascular Medicine, Nagasaki University Hospital, Nagasaki, Japan; ^4^ Graduate School of Frontier Sciences, Department of Computational Biology and Medical Sciences, The University of Tokyo, Tokyo, Japan; ^5^ AIDS Research Center, National Institute of Infectious Diseases, Tokyo, Japan; ^6^ Department of Hepatobiliary–pancreatic Surgery, Graduate School of Medicine, The University of Tokyo, Tokyo, Japan; ^7^ National Center for Global Health and Medicine, Tokyo, Japan

**Keywords:** pancreatic cancer, Glypican-1, EVI1, KRAS

## Abstract

Glypican-1 (GPC1) protein in exosomes was recently identified as a biomarker for the early detection of pancreatic ductal adenocarcinoma (PDAC). Immunohistochemical analyses and *in vitro* assays were conducted to assess the usefulness of GPC1 as a PDAC biomarker, to reveal the biological role of GPC1 in pancreatic carcinogenesis, and to ascertain the regulation mechanism of GPC1. An aberrant overexpression of GPC1 protein which is usually absent in normal pancreatic duct, was a widespread marker across the full spectrum of human PDAC precursors, PDAC, and pancreatic cancerous stroma. In intraductal papillary-mucinous neoplasms (IPMNs), GPC1 tended to be positive in gastric-type IPMN. *KRAS* mutations were found in all GPC1-positive IPMN cases and in one-third of GPC1-negative IPMN cases. In pancreatic cell lines, GPC1 depletion caused remarkable inhibition of cell growth and migration, suggesting its oncogenic roles. GPC1 depletion upregulated the molecules associated with cell cycle arrest in pancreatic cell lines. Furthermore, KRAS and ecotropic viral integration site 1 (EVI1) oncoprotein upregulated GPC1 expression. In a clinical cohort, GPC1 overexpression was not correlated with pancreatic cancer prognosis. Taken together, these findings suggest the necessity of establishing a threshold of GPC1 value for detecting pancreatic malignancy because GPC1 is overexpressed even in low-grade PDAC precursors which do not always become malignant. Our study also reveals a new aspect of pancreatic carcinogenesis: KRAS and EVI1, two important molecules in early phases of pancreatic carcinogenesis, positively regulate GPC1 expression and likely promote pancreatic carcinogenesis.

## INTRODUCTION

Pancreatic ductal adenocarcinoma (PDAC) elicits prognoses that are among the worst of all cancers. Multistep pancreatic carcinogenesis has been proposed from precursor epithelial lesions such as pancreatic intraepithelial neoplasia (PanINs), intraductal papillary mucinous neoplasms (IPMNs), and mucinous cystic neoplasms (MCNs) [1]. At the molecular level, activating mutations in *KRAS* are early and universal events. They are followed chronologically by inactivating mutations in *CDKN2A*, *TP53*, and *SMAD4* [2]. This stepwise theory is supported by a genetic progression model of pancreatic carcinogenesis that gives rise to formation of an infiltrating cancer. A computational model that incorporates the number of somatic alteration, driver versus passenger events, founder versus progressor alterations, and relative proliferation rates of cells yielded an average of 11.7 years from initiation to development of the parental clone in pancreatic carcinogenesis [2–4]. Development of invasive pancreatic carcinoma occurs only after a long latency period. Therefore, detecting pancreatic precancerous (non-invasive) lesions during this latency period is necessary to improve the PDAC prognosis because early stage pancreatic cancers are associated with better survival [5]. However, almost no useful clinical tools are available to enable early detection. The primary requisites verify the chronological change occurring in the early phase of pancreatic carcinogenesis and suggest a new modality including a valid tumor marker to treat this intractable disease.

For pancreatic cancer detection, several serum markers have been used, such as CA19-9, carcinoembryonic antigen, DU-PAN-2, or Span-1. However, these markers do not appear to be optimal for pancreatic cancer detection. Recently, Melo *et al.* reported that glypican-1 (GPC1), a membrane-anchored proteoglycan molecule enriching circulating exosomes (GPC1+ crExos), might be useful as a non-invasive diagnostic and screening tool to detect early pancreatic cancer and to surpass conventionally used tumor markers [6]. GPC1 was detected even in cases having only pancreatic precancerous lesion. It was not detected in non-neoplastic lesions such as chronic pancreatitis. The expression level of GPC1 decreased considerably after surgical resection of pancreatic cancer. They also discovered that the mutant *KRAS* transcript was detected only within the GPC1+ crExos. Nevertheless, not all precancerous lesions developed into PDAC. The number of PanIN, low-grade was increased during aging or in chronic pancreatitis. Whether GPC1 was a true cancer marker remains to be elucidated. Furthermore, no explanation exists for some cases in which a drop of GPC1 value was insufficient after surgical resection of pancreatic cancer.

Earlier reports have described the tumor-promoting role of GPC1 in several cancers. In glioma or breast cancer, GPC1 is frequently overexpressed. It modulates the mitogenic effects of heparin-binding growth factors [7–9]. In pancreatic cancer, GPC1 is physiologically necessary for mitogenic signaling of FGF2 and HB-EGF. It modulates TGF-β-dependent signaling and angiogenic and metastatic potential [10–14]. Furthermore, GPC1 enhances tumor growth, angiogenesis, and invasion in an oncogenic *KRAS*-driven mouse model of PDAC [15]. In a clinical cohort, GPC1 was found to be related to perineural invasion and poor prognosis [16]. If GPC1 truly serves as a tumor-promoting role in pancreatic cancer as described in earlier reports of the literature, then elucidating the regulation mechanism of GPC1 expression is expected to provide insights to support inhibition of pancreatic carcinogenesis.

We attempted to resolve the two issues as explained below. First, we examined the expression of GPC1 in human tissue immunohistochemically and ascertained the stage of the pancreatic precancerous lesion at which GPC1 became expressed and whether GPC1 was specific for pancreatic ductal precancerous and cancerous lesion, or not. We also examined the relation between expression of GPC1 and *KRAS* mutation status. Then, we evaluated the existence of tumor-promoting effects of GPC1 in pancreatic cancer *in vitro*. Results show the regulation mechanism of expression of GPC1. Results highlight the expression pattern of GPC1 in precancerous lesions compared with the gastric epithelial metaplasia marker, especially transcriptional factor ecotropic virus integration site 1 (EVI1), which occurs in the early phase of pancreatic carcinogenesis [17–21].

## RESULTS

### Widespread overexpression of GPC1 protein in pancreatic neoplasms

**Figure 1:**
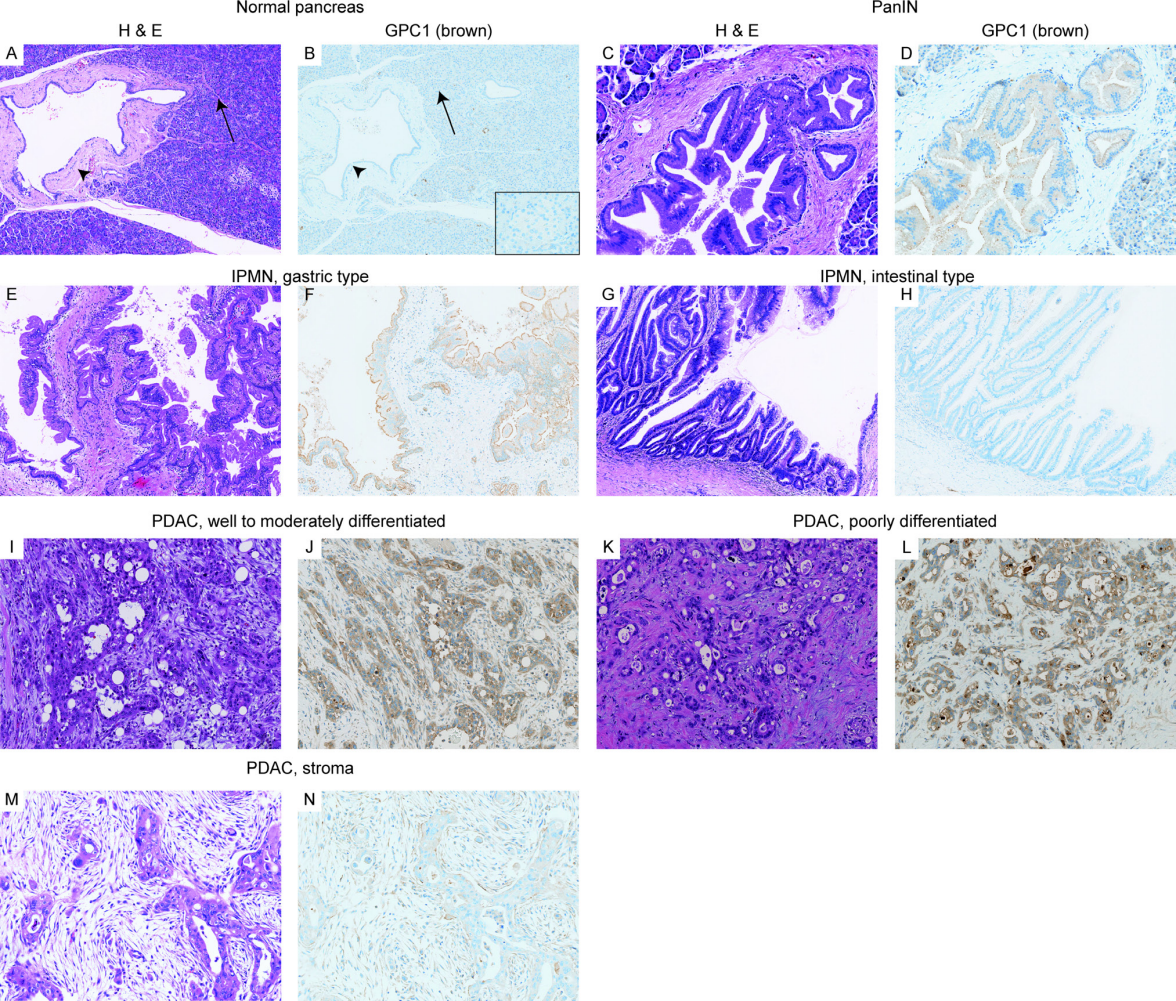
Expression of GPC1 protein in human pancreatic tissues (**A**–**N**) Immunohistochemical analysis of GPC1 in pancreatic tissue (H&E staining; GPC1 immunostaining). (A–B) Non-neoplastic pancreas. Normal pancreatic ducts (black arrowhead) and islet (inset) do not express GPC1. Acini (black arrow) weakly express GPC1. (C–D) PanINs. GPC1 is expressed in the PanIN-1 lesion (low-grade PanIN). (E–H) IPMNs. In the IPMN lesion, GPC1 is expressed moderately in gastric-type IPMN (E–F), but is not expressed in intestinal-type IPMN (G–H). (I–N) PDACs. In well to moderately differentiated PDACs (I–J) and poorly differentiated PDACs (K–L), GPC1 is expressed strongly in cytoplasm and membrane. GPC1 is also expressed in stroma of PDAC (M–N).

To evaluate the expression pattern of GPC1 in pancreatic neoplasms, we used immunohistochemical analysis of human pancreatic tissue, as presented in Table 1 and Figure 1. In normal pancreatic tissue, pancreatic ductal epithelial cells and endocrine cells did not express GPC1. Pancreatic acini weakly expressed GPC1 (Figure 1A, 1B). Normal gastrointestinal epithelium was used as positive controls because we confirmed strong expression of GPC1 in normal gastrointestinal epithelium. Ductal metaplasia of acinar cells also showed weak or absent expression of GPC1. In contrast, GPC1 was expressed diffusely in the cytoplasm and membrane of neoplastic cells in PDAC precursors, PanIN (87.0%, 20/23) (PanIN-1: Figure 1C, 1D). GPC1 was expressed from low-grade PanIN to high-grade PanIN. In IPMN, GPC1 was expressed in about half of the cases (58.1%, 79/136). In many cases of gastric-type IPMNs, oncocytic-type IPMNs, and pancreatobiliary-type IPMNs, GPC1 was expressed diffusely in the cytoplasm and membrane (Figure 1E, 1F). However, GPC1 was not expressed in most cases of intestinal-IPMNs (Figure 1G, 1H). In MCNs, 25% of cases expressed GPC1. In PDACs, about 70% of cases demonstrated GPC1 overexpression. The expression pattern was not correlated with the degree of differentiation (Figure 1I, 1J, 1K, 1L). In PDACs, about 50% of cases demonstrated GPC1 expression in the stromal cells surrounding the pancreatic cancers (Figure 1M, 1N). No direct correlation of GPC1 expression was found between pancreatic cancer cells and pancreatic cancer stroma. We also examined the expression of GPC1 in other pancreatic neoplasms. In acinar cell carcinomas, no GPC1 expression was observed (0/5). In solid-papillary tumors, GPC1 was very weakly expressed in about 60% of case (5/8). In neuroendocrine tumors, about 80% of cases showed GPC1 expression (14/17). For chronic pancreatitis, we observed several cases of PanIN, low-grade, with weak to moderate GPC1 expression (15 of 20 chronic pancreatitis cases).

**Table 1:** GPC1 expression in normal pancreas and pancreatic neoplasm

Histology	GPC1	
	Positive rate	Expression score (0/1/2/3/4/5)
Normal pancreatic duct	Negative	
Normal pancreatic acinus	Weakly positive	
Normal pancreatic islet	Negative	
Ductal metaplsia of acinar cells	Negative	
		
PanIN		
PanIN-1	6/8 (75%)	2/0/5/1/0/0
PanIN-2	10/11 (90.9%)	1/0/10/0/0/0
PanIN-3	4/4 (100%)	0/0/4/0/0/0
		
IPMN		
Low-grade dysplasia	42/75 (56.0%)	23/10/26/3/10/3
High-grade dysplasia	12/26 (46.1%)	10/4/10/0/2/0
Carcinoma, invasive	25/35 (71.4%)	5/5/10/6/9/0
		
Gastric-type	55/91 (60.4%)	21/15/35/4/13/3
Intestinal-type	4/21 (19.0%)	15/2/4/0/0
Oncocytic-type	3/4 (75.0%)	1/0/0/1/2/0
Pancreatobiliary-type	11/14 (78.6%)	1/2/5/3/3/0
		
MCN	3/12 (25.0%)	
		
PDAC		
Neoplastic cells		
Well-differentiated	52/71 (73.2%)	7/12/50/2/0/0
Moderately differentiated	124/182 (68.1%)	24/34/109/12/3/0
Poorly differentiated	41/58 (70.1%)	7/10/36/1/3/1
Stroma		
Well-differentiated	35/71 (49.3%)	
Moderately differentiated	97/182 (53.3%)	
Poorly differentiated	24/58 (41.4%)	
		
Acinar cell carcinoma	0/5 (0%)	
Solid-papillary tumor	5/8 (62.5%)	
Neuroendocrine tumor	14/17 (82.4%)	

### Overexpression of GPC1 protein correlates with overexpression of EVI1 in pancreatic neoplasms and with *KRAS* mutation in gastric-type pancreatic neoplasms

The results presented above demonstrated that GPC1 is expressed from precancerous lesions such as those of PanIN or IPMN in pancreatic carcinogenesis. Actually, in pancreatic carcinogenesis, the acquisition of the extrapancreatic foregut or gastric epithelial markers occurs during an early phase of pancreatic carcinogenesis. Based on these morphological and phenotypical findings, we found previously that overexpression of EVI1 oncoprotein marks the full spectrum of human PDAC precursors and PDAC [21]. Then we examined the expression pattern of GPC1 and EVI1 in precancerous lesions of the pancreas. In IPMN, the expression pattern of GPC1 was well correlated with the expression pattern of EVI1 (Table 2, *p* < .05). The same tendency was observed similarly for PanIN.

**Table 2:** The relation between EVI1 expression and GPC1 expression in IPMN and PanIN, and KRAS status and GPC1 expression in IPMN

		GPC1		
		PanIN		
		Positive	Negative	
EVI1	Positive	19	3	
	Negative	0	0	
		IPMN		
		Positive	Negative	*p*-value
EVI1	Positive	88	70	0.0159^*^
	Negative	2	9	

GPC1				
Positive		Negative		
*KRAS* status	IPMN subtype	*KRAS* status	IPMN subtype	
G12V	Pancreatobiliary	Wild-type	Oncocytic	
G12V	Pancreatobiliary	Wild-type	Pancreatobiliary	
G12V	Pancreatobiliary	Wild-type	Intestinal	
G12R	Gastric	Wild-type	Intestinal	
G12V	Gastric	Wild-type	Intestinal	
G12V	Gastric	Wild-type	Intestinal	
G12V	Gastric	Wild-type	Intestinal	
G12D	Gastric	G12D	Intestinal	
G12D	Gastric	G12S	Intestinal	
G12D	Gastric	G12D	Intestinal	
		G12V	Intestinal	
		G12D	Intestinal	

Considering a report by Melo *et al.* describing that GPC1-positive circulating exosomes in pancreatic cancer patients contain oncogenic *KRAS*^G12D^, we examined the relation between GPC1 expression and *KRAS* status (Table 2). All GPC1-positive cases possessed *KRAS*^G12V^, *KRAS*^G12D^, or *KRAS*^G12R^ mutation and all those cases were pancreatobiliary-type and gastric-type IPMN. The GPC1-negative cases possessed both wild-type *KRAS* and mutant *KRAS*. The GPC1-negative cases with mutant *KRAS* were all intestinal-type IPMN. Our study comparing the *KRAS* gene sequence and GPC1 expression used a different experiment system from that of the study of Melo *et al*., which compared KRAS mRNA expression within the exosome and GPC1 expression. However, our study results differed only slightly from those reported by Melo *et al.*in that mutant KRAS was detected within GPC1-negative cases.

### EVI1 regulates GPC1 expression in non-neoplastic pancreatic duct cell lines

**Figure 2:**
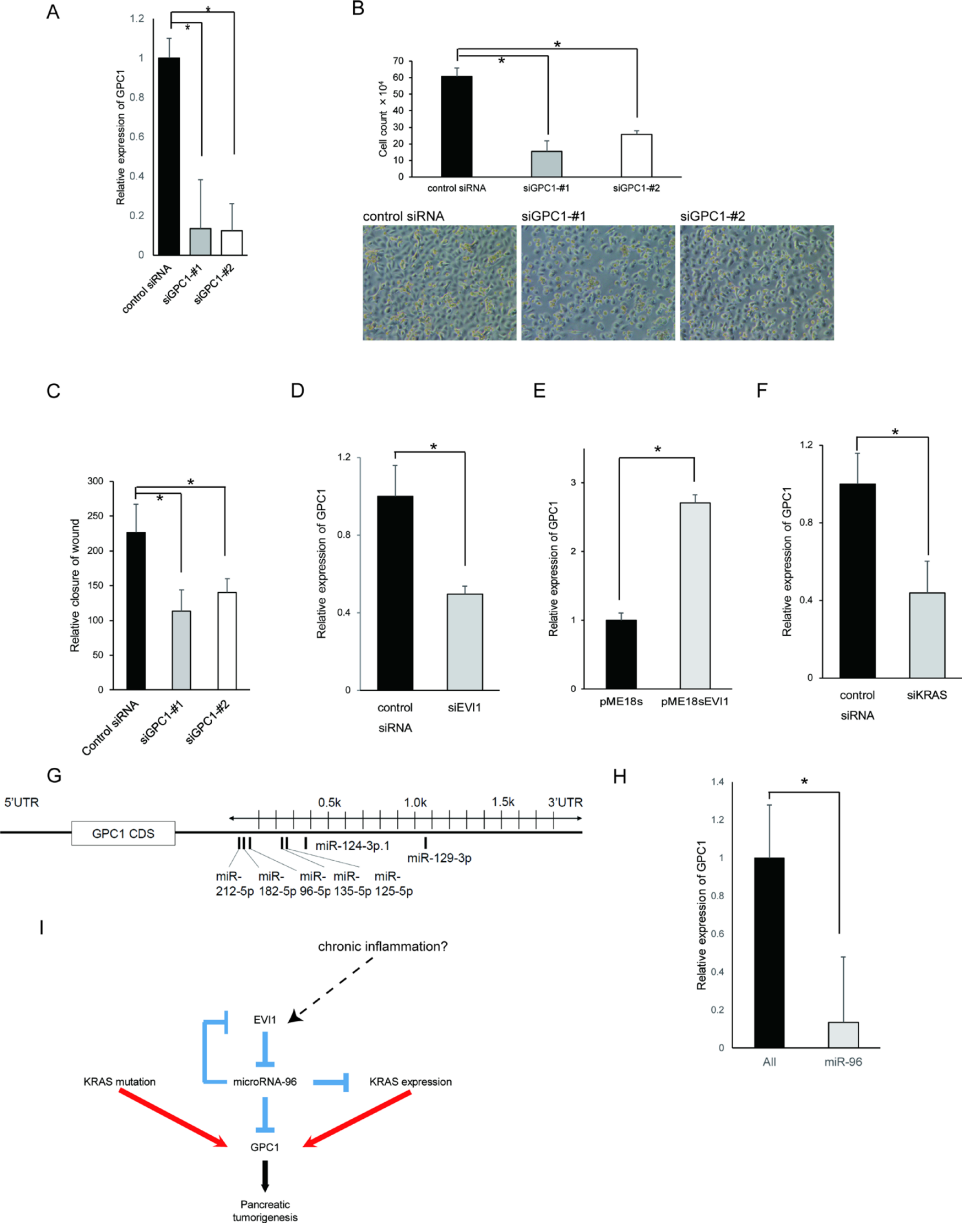
EVI1 regulates GPC1 expression in HPDE cell lines (**A**) Knockdown efficacies for two siRNA for GPC1 were confirmed in HPDE cell lines. (**B**) Effects of GPC1 knockdown on proliferation of HPDE cell lines. Growth assay was conducted in 6-well plates and the numbers of cells were counted with trypan blue staining. Experiments were conducted in triplicate. ^*^p < .05. The photographs show cell density. (**C**) Effects of GPC1 knockdown on cellular migration of HPDE cell lines. Migration activity was examined in normal medium in HPDE cell lines. Experiments were conducted in triplicate. ^*^p < .05. (**D**) Downregulation of GPC1 mRNA expression by EVI1 knockdown. After transfection with control siRNA or siEVI1 in HPDE cell lines, qRT-PCR assay was performed. (**E**) Upregulation of GPC1 mRNA expression by EVI1 overexpression. After transfection with pME18s plasmid or pME18s-EVI1 plasmid in HPDE cell lines, qRT-PCR assay was performed. ^*^p < .05. (**F**) Downregulation of GPC1 mRNA expression by KRAS knockdown. After transfection with control siRNA or siKRAS in HPDE cell lines, qRT-PCR assay was performed. (**G**) Schematic diagram of human GPC1 3′UTR and potential miRNA target sites. (**H**) miR-96 inhibits GPC1 expression. After transfection with negative control or synthetic miRNAs (20 nM) in HPDE cell lines, qRT-PCR assay was performed. ^*^p < .05. (**I**) Summary of relation between EVI1, KRAS, and GPC1 in pancreatic carcinogenesis. Red lines represent promotion and blue lines represent inhibition. The speculative relation was expressed as a dot line.

GPC1 was found to be widely expressed in PDAC precursors (Table 1). Therefore, we hypothesized that GPC1 promoted pancreatic carcinogenesis, even from a precancerous stage. Using an immortalized human pancreatic ductal epithelial cell line, HPDE cell lines, as a model of precancerous pancreatic cells, we examined the role of GPC1 by *in vitro* assays. Two siRNAs against GPC1 were designed and the knockdown efficacies for these two siRNA were confirmed (Figure 2A). We confirmed that similar results were obtained for both siRNAs. In HPDE cell lines, downregulation of GPC1 by siRNA inhibited cell proliferation and cell migration considerably (Figure 2B, 2C). These results indicate that GPC1 promotes carcinogenesis even in non-cancerous pancreatic cells and suggest that control of GPC1 expression is important for pancreatic stepwise carcinogenesis.

We investigated the regulation of GPC1 expression. Regarding correlation between the expression patterns of EVI1 and GPC1 in pancreatic precancerous lesions, we hypothesized that EVI1 regulates GPC1 expression. Results show that downregulation of EVI1 by siRNA significantly decreased the expression level of GPC1 mRNA in HPDE cell lines (Figure 2D). Overexpression of EVI1 by pME18s plasmid transfection significantly upregulated the expression level of GPC1 mRNA in HPDE cell lines (Figure 2E). These results indicate that EVI1 might regulate GPC1 expression.

We also examined the relation between KRAS and GPC1 because GPC1 expression was partly correlated with *KRAS* status (Table 2) and because KRAS is extremely important in pancreatic precancerous lesions. The expression level of GPC1 was downregulated by KRAS knockdown in HPDE cell lines (Figure 2F).

EVI1 is a transcriptional factor. Therefore, we examined whether EVI1 can bind directly to GPC1 promoter region and promote GPC1 expression through *in silico* analysis. However, we were unable to find the EVI1 binding site at the promoter region of GPC1. We next explored the involvement of microRNAs (miRNAs) in EVI1-mediated GPC1 regulation. miRNAs act as post-transcriptional regulators of gene expression and elicit tumor-suppressive and oncogenic functions, respectively, by targeting oncogenes and tumor suppressors [22–23]. Several miRNAs such as miR-96, -124, -125, -129, -135, -182, and -212 were predicted to target the GPC1 3′-untranslated region (3′UTR) using a number of target prediction algorithms (Figure 2G). We compared these miRNAs to previously reported miRNAs associated with pancreatic cancer. Previous profiling studies showed that miR-96 was downregulated and miR-212 was upregulated on PDAC tissues [24]. Almost all miRNAs negatively regulate gene expression and GPC1 was upregulated in pancreatic cancer. Therefore, we surmised that miRNA that regulates expression of GPC1 is downregulated in pancreatic cancer. We specifically examined miR-96. Results revealed that miR-96 introduction significantly suppressed the levels of GPC1 mRNA expression in HPDE cell lines (Figure 2H).

Figure 2I presents a summary of GPC1 regulation by EVI1 and KRAS in HPDE cell lines. miR-96 is known as a negative regulator of KRAS. Our earlier report described that EVI1 located at the upstream of miR-96 in pancreatic cells [21].

### EVI1 modulates the oncogenic role of GPC1 in pancreatic cancer

Considering that GPC1 was expressed in about 70% of PDAC cases (Table 1) and because several previous reports have described the oncogenic roles of glypicans in cancer, for example, GPC3 associated with the progression of malignant tumors of several types, including mesotheliomas and ovarian cancer, or a role for GPC1 in pancreatic cancer progression, we analyzed the role of GPC1 in pancreatic cancers using *in vitro* assays.

**Figure 3:**
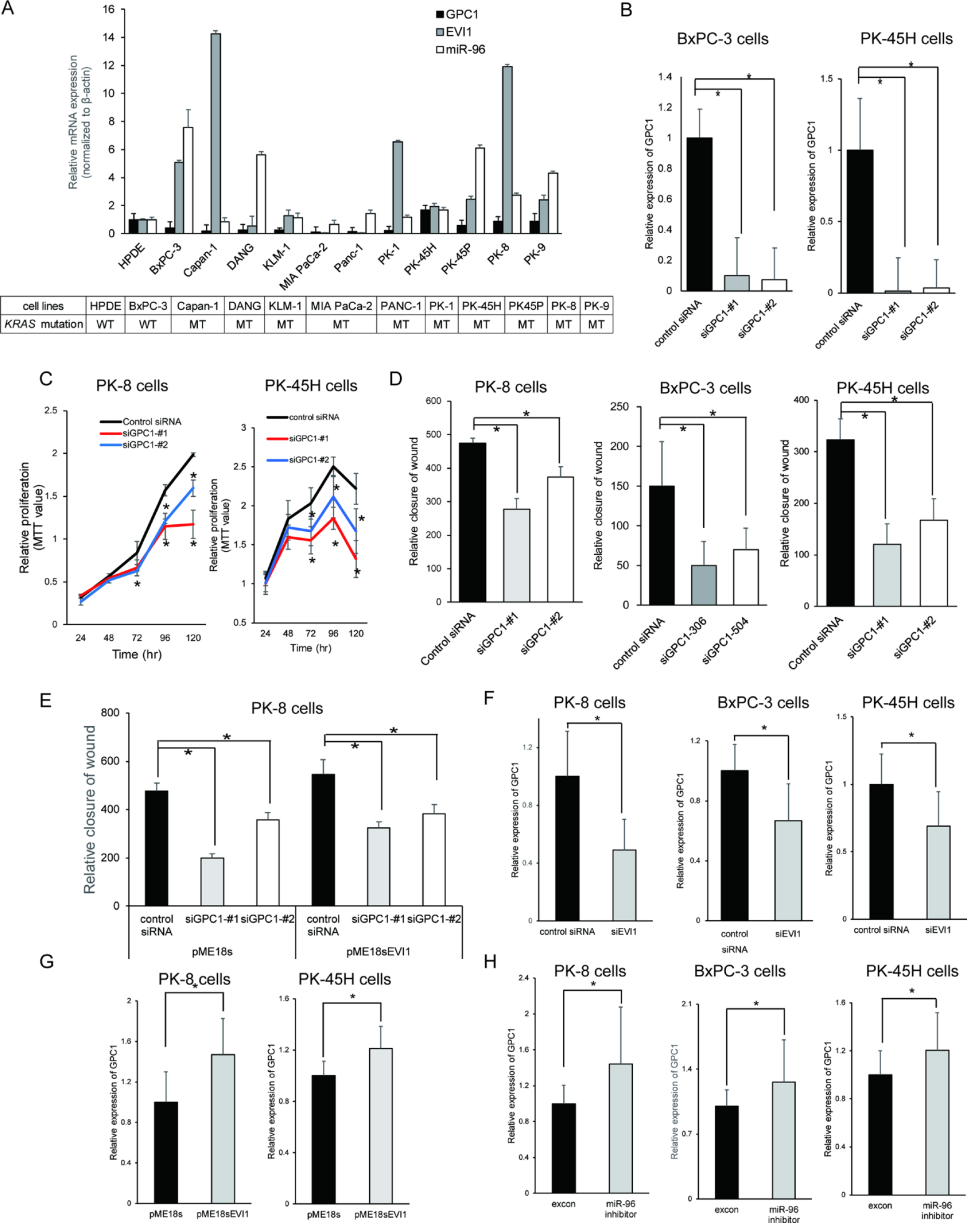
EVI1 modulates the oncogenic role of GPC1 in pancreatic cancer (**A**) Expression of GPC1 mRNA, EVI1 mRNA and mature miR-96 and *KRAS* mutation status in several pancreatic-lineage cell lines. Effects of GPC1 knockdown on cellular migration of pancreatic cancer cell lines. (**B**) Knockdown efficacies for two siRNA for GPC1 were confirmed in pancreatic cell lines. (**C**) Effects of EVI1 knockdown on proliferation of GPC1-positive pancreatic cancer cell lines (PK-8 and PK-45H cell lines). Growth assay was conducted in 96-well plates where cells were plated at 3,000 cells per well and grown in 10% FBS medium. Experiments were conducted in triplicate. ^*^p < .05. (**D**) Migration activity was examined in 10% FBS medium in pancreatic cell lines (PK-8, BxPC-3 and PK-45H cell lines). Experiments were conducted in triplicate. ^*^p < .05. (**E**) Overexpression of EVI1 attenuates migration inhibition by GPC1 knockdown in PK-8 cell lines. PK-8 cell lines were transfected with control siRNA or siGPC1, transfected with pME18s or pME18s-EVI1 plasmid and then wound healing assay. ^*^p < .05. (**F**) Downregulation of GPC1 mRNA expression by EVI1 knockdown. After transfection with control siRNA or siEVI1 in pancreatic cell lines (PK-8, BxPC-3 and PK-45H cell lines), qRT-PCR assay was performed. ^*^p < .05. (**G**) Upregulation of GPC1 mRNA expression by EVI1 overexpression. After transfection with pME18s plasmid or pME18s-EVI1 plasmid in PK-8 cell lines or PK-45H cell lines, qRT-PCR assay was performed. ^*^p < .05. (**H**) Upregulation of GPC1 mRNA expression by miR-96 inhibitor. After transfection with negative control or miR-96 inhibitor in pancreatic cell lines (PK-8, BxPC-3 and PK-45H cell lines), qRT-PCR assay was performed. ^*^p < .05.

We examined the expression of GPC1 using real-time quantitative reverse transcription PCR (qRT-PCR) in 11 pancreatic cancer cell lines (BxPC-3, Capan-1, DANG, KLM-1, MIA PaCa-2, PANC-1, PK-1, PK-45H, PK-45P, PK-8, PK-9 cell lines) and HPDE cell lines (Figure 3A). A correlative tendency between expression of GPC1, EVI1 and miR-96 was found. We decided to use represented pancreatic cancer cell lines PK-8, PK-45H and BxPC-3 highly expressing GPC1. The knockdown efficacies of siRNA for GPC1 were confirmed in pancreatic cancer cell lines (Figure 3B). In these pancreatic cancer cell lines, downregulation of GPC1 by siRNA significantly suppressed cell proliferation (Figure 3C) and inhibited cell migration (Figure 3D). These results indicate that GPC1 plays an oncogenic role in pancreatic cancer.

The relation between EVI1/miR-96 and GPC1 found in HPDE cell lines was further confirmed in pancreatic cancer cell lines. In pancreatic cancer cell lines, overexpression of EVI1 by plasmid transfection exhibited a tendency to attenuate migration inhibition by silencing of GPC1 in PK-8 cell lines (Figure 3E). Downregulation of EVI1 by siRNA decreased the expression level of GPC1 mRNA significantly, whereas overexpression of EVI1 by pME18s plasmid transfection significantly upregulated the expression level of GPC1 mRNA and miR-96 inhibition upregulated the levels of GPC1 mRNA expression (Figure 3F–3H). By gene expression microarray analysis, we confirmed a positive correlation of the respective mRNA expression patterns of GPC1-depleted PK-45H cell lines and EVI1-depleted PK-45H cell lines (Supplementary Figure 1A). Gene ontology analysis about common genes which were downregulated both in siEVI1 group and siGPC1 group revealed that genes related to cell proliferation were enriched (Supplementary Figure 1B, 1C).

These results suggest that EVI1 is at least partially involved in GPC1-driven pancreatic oncogenicity, not only in the precancerous stage but also up to the cancerous stage.

### GPC1 function in PDAC gene expression signature

**Figure 4:**
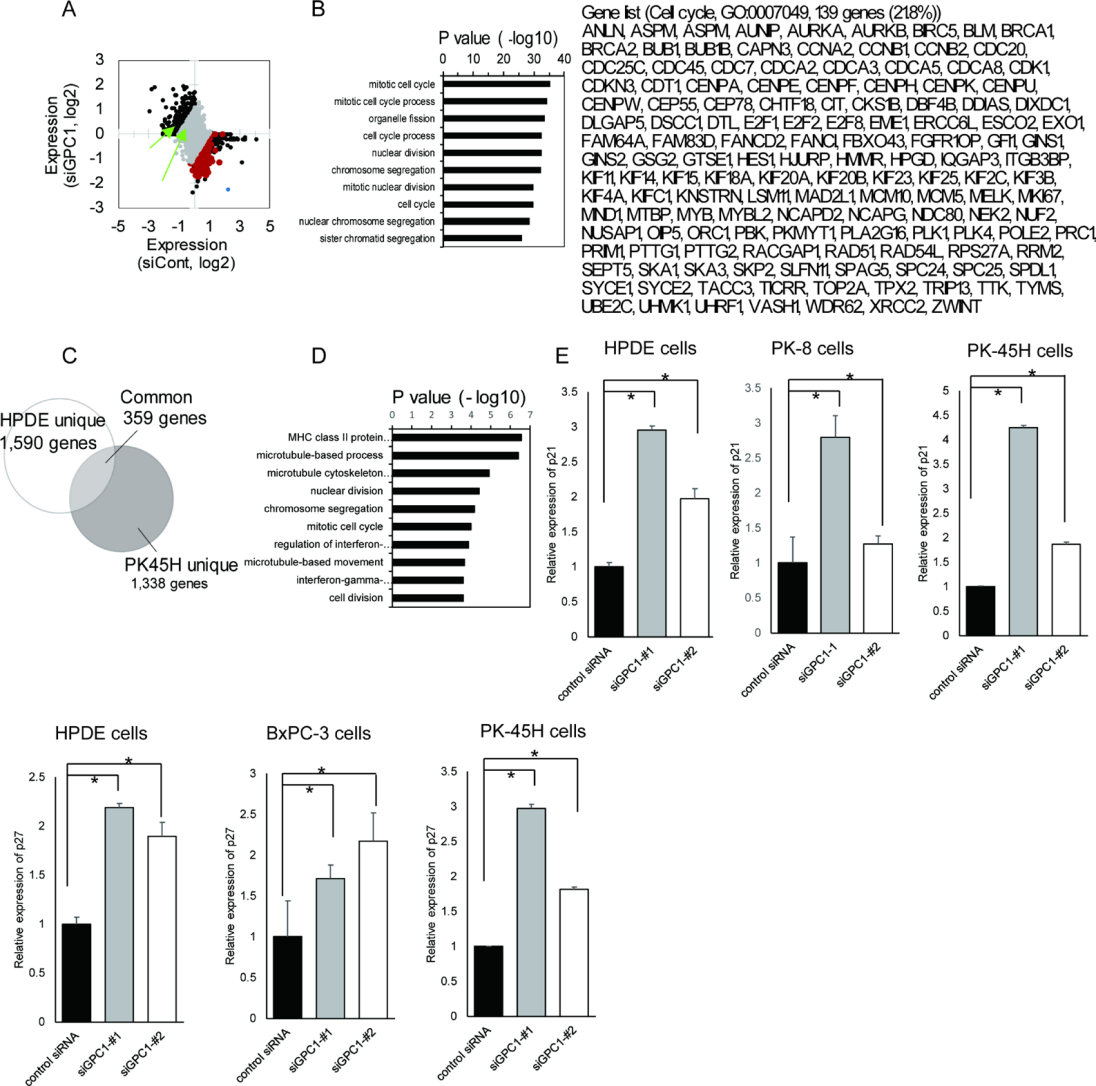
Function of EVI1-GPC1 axis in PDAC carcinogenesis (**A**) Scatter plot showing all gene expression pattern in PK-45H cell lines with control siRNA (x-axis) and siGPC1 (y-axis). Averaged values from two independent siRNAs targeting GPC1 are shown. Black dots represent significantly changed genes by siGPC1 (fold-change (FC) >2 or FC<-2). Genes which are related to cell cycle regulation and downregulated in siGPC1 group are shown by red (shown in Figure 4B). Green arrows indicated the p21 and p27 mRNA expression. The expression of GPC1 is shown as blue dots. (**B**) Gene ontology analysis of GPC1 target genes in PK-45H cell lines (660 genes which were downregulated in siGPC1 group). Significant GO terms and their *P* values are shown by a bar graph. Genes involved in cell cycle regulation are shown on the right. (**C**) Venn diagram showing genes significantly downregulated by siGPC1 in HPDE cell lines (white circle, FC<-1.5) and in PK-45H cell lines (gray circle, FC<-1.5). (**D**) Gene ontology analysis of common 359 genes which were downregulation in siGPC1 group both in HPDE cell lines and PK-45H cell lines). Significant GO terms and their *P* values are shown by a bar graph. (**E**) Upregulation of p21 and p27 mRNA expression by GPC1 knockdown in HPDE cell lines and several pancreatic cancer cell lines. ^*^p < .05.

To assess the functional involvement of EVI1-GPC1 axis in PDAC carcinogenesis, we performed whole expression analysis in pancreatic cancer cell models (*KRAS*-mutated PK-45H cell lines) with siGPC1 or siEVI1 using gene expression microarray analysis. Results show that GPC1 expression was diminished almost completely by both siGPC1-#1 and siGPC1-#2. The GPC1-depleted PK-45H cell lines showed differential transcriptome compared with the parental control cell lines (Figure 4A). Results also show that genes involved in cell cycle progression are enriched considerably in the downregulated genes in GPC1-depleted PK-45H cell lines. Gene ontology analysis revealed that GPC1 plays a role for induction of genes contributing to cell cycle progression (Figure 4B) which is consistent with results of cell proliferation assay (Figure 3C). We also confirmed that a similar molecular process were enriched in GPC1-depleted PK-45H cell lines and GPC1-depleted HPDE cell lines (Figure 4C, 4D). This screening also identified CDKN1A (p21) and CDKN1B (p27) as upregulated genes by GPC1-depletion in HPDE cell lines and pancreatic cancer cell lines (Figure 4E).

### GPC1 expression and clinicopathological characteristics

**Figure 5:**
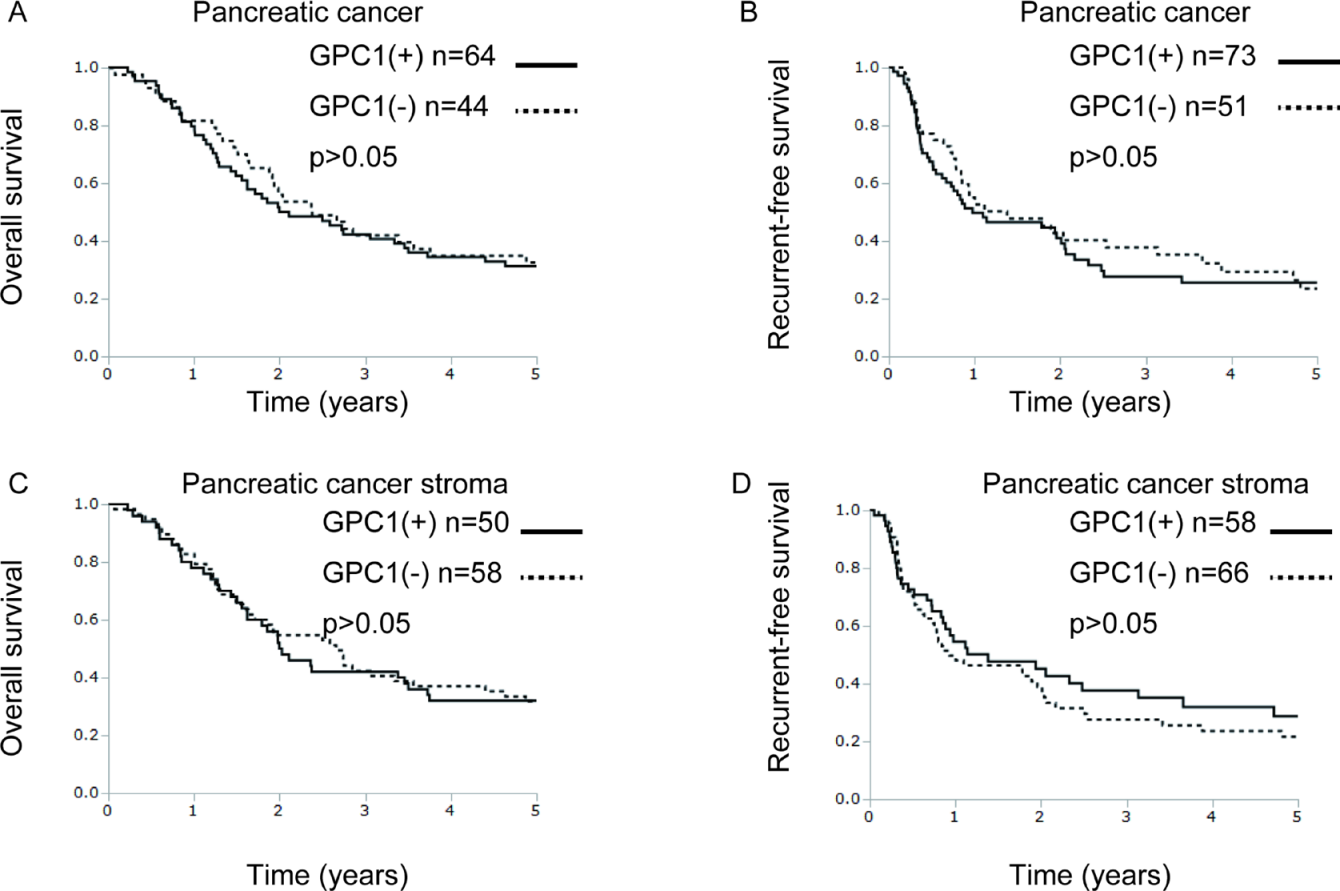
GPC1 protein expression is not correlated with overall survival or recurrence-free survival in pancreatic cancer patients Kaplan–Meier curves of overall survival and disease-free survival of the pancreatic cancer cases were assessed according to the IHC score of GPC1. (**A**–**B**) In pancreatic cancers, no significant difference was found between GPC1-positive groups and GPC1-negative groups for overall survival (A) or for recurrence-free survival (B). (**C**–**D**) In pancreatic cancers, no significant difference was found between stromal GPC1-positive groups and stromal GPC1-negative groups for overall survival (C) or for recurrence-free survival (D).

We examined the relation between the expression of GPC1 and clinicopathological characteristics and prognosis in PDAC patients of The University of Tokyo (Table 3 and Figure 5). Age, tumor location, and vascular invasion were associated with expression of GPC1 in a clinical cohort (Table 3). The overall survival and disease-free survival were not correlated with GPC1 expression in pancreatic cancer cells or in pancreatic cancerous stromal cells in our clinical cohorts (Figure 5).

**Table 3:** Relation between the expression of GPC1 and clinicopathological characteristics

		GPC1		*p* value	
		Positive	Negative	chi-square test	Fisher’s exact test
Sex					
	Male	127	66	0.065	0.0716
	Female	85	27		
Age (yr)					
	< 60	41	30	0.0140^*^	0.0183^*^
	≧ 60	171	63		
Tumor location					
	Head	120	76	0.0001^*^	
	Body-tail	81	16		
	Whole pancreas	11	1		
Tumor size (cm)					
	< 2	18	12	0.3015	
	2 to 5	154	68		
	> 5	36	11		
TNM stage					
	I–II	201	90	0.4508	0.563
	III–IV	11	3		
Histological differentiation					
	Well	51	19	0.525	
	moderately	122	57		
	Poorly	17	39		
Lymph node involvement					
	(+)	133	61	0.6332	0.6987
	(−)	79	32		
Lymph vessel invasion					
	(+)	137	63	0.279	0.6947
	(−)	75	30		
Vascular invasion					
	(+)	199	75	0.0004^*^	0.0008^*^
	(−)	13	18		
Perineural invasion					
	(+)	198	83	0.7137	0.8051
	(−)	14	7		

## DISCUSSION

GPC1 was expressed in almost all pancreatic neoplastic lesions, but not in the normal pancreatic duct by immunohistochemical analysis in the present study. This expression pattern of GPC1 supports the notion presented in a report by Melo *et al.* that GPC1+crExos is useful as a screening tool for early pancreatic cancer. However, our data also show that GPC1 was expressed from low-grade precancerous lesions of the pancreas, which did not always develop into carcinoma. These results indicate the need to set a reasonable cutoff value if we are to use GPC1+crExos as a pancreatic cancer screening tool. Melo *et al.* reported that GPC1 was not decreased after surgical resection in some cases. This phenomenon might be explained by incomplete removal of carcinoma of the remnant pancreas and by low-grade PanINs of the remnant pancreas. The following findings that should also be examined. GPC1 was not expressed in intestinal-type pancreatic neoplasms, which tend to occur in main pancreatic duct and which have the potential to transform to invasive carcinoma [1]. In addition, GPC1 was expressed in pancreatic ductal neoplasms, and also in pancreatic neuroendocrine tumors or solid-papillary tumors.

From *in vitro* assays, we found that GPC1 committed to cellular proliferation or migration, that EVI1, KRAS and/or miR-96 regulated expression of GPC1, and that EVI1 modulated the oncogenic role of GPC1 in both pancreatic noncancerous cell lines and pancreatic cancer cell lines. Our earlier report described that EVI1 binds to the potential binding site around miR-96 in pancreatic cancer cells and that miR-96 has a potential binding site at 3′-UTR of KRAS in pancreatic cancer cells [21]. Considering that no EVI1 direct binding site was found at the promoter region of GPC1 and that downregulation of miR-96 was reported in pancreatic cancer [25], it might be speculated that EVI1 regulated the expression of GPC1 through miR-96 and that overexpression of KRAS or *KRAS* mutation also adjusted the expression level of GPC1 in pancreatic carcinogenesis (Figure 2I). These assays revealed the GPC1-modulatory effects by EVI1/KRAS/miR-96 were observed more clearly in precancerous HPDE cell lines than in pancreatic cancer cell lines despite comparable GPC1 mRNA levels (Figure 3A, e,g, PK-8 and PK-45H), implying that this axis holds more importance in the earlier stages of the onset of carcinogenesis. The lack of clear correlation of GPC1 expression levels with pancreatic cancer prognosis (Figure 5) is also in line with this notion. However, the results showing that EVI1 can rescue the oncogenic role of GPC1 in PK-8 cell lines might support the presence of EVI1/KRAS/miR-96-GPC1 axis, even in the cancerous stage of pancreatic carcinogenesis. The relative contribution of GPC1 expression dysregulation to carcinogenesis as compared with EVI1/miR-96/KRAS dysregulation remains to be elucidated. It is noteworthy that alternative mechanisms such as methylation of GPC1 promoter regions or GPC1 acetylation might concomitantly regulate expression of GPC1.

Through mutational analyses of human pancreatic neoplasm and establishment of mutant *KRAS*-driven pancreatic cancer mouse models, researchers have regarded oncogenic *KRAS* as crucially important for pancreatic carcinogenesis. However, the findings that only one-third of human PanIN-1 possess oncogenic *KRAS* and that only a small subset of cells with mutant *KRAS* expression can develop into PanINs in mouse models do not indicate *KRAS* mutation as absolutely responsible for the initiation of PDAC precursors [26–28]. These observations give rise to the hypothesis that molecular mechanisms other than *KRAS* mutation contribute to the expansion of PanIN cells and promote pancreatic carcinogenesis. Our results suggest that GPC1 overexpression is correlated not with *KRAS* mutation but with the gastric phenotype. Consequently, GPC1 overexpression might contribute to pancreatic carcinogenesis both dependently on and independently of *KRAS* mutation.

An earlier report described that GPC1 overexpression is correlated with poor prognosis in pancreatic cancer [16], but our results did not indicate differences in prognoses between GPC1-positive cases and GPC1-negative cases. Considering that GPC1 was expressed from precancerous lesions of the pancreas, GPC1 might not be involved in the malignant potential of the advanced phase of pancreatic cancer. Rather, it might be involved in early carcinogenesis process by which the normal pancreatic ductal epithelium gradually becomes the carcinogenic epithelium. For this reason, in cases of advanced pancreatic cancer, the pathogenic role of GPC1 might not be a central one.

This study has certain limitations. One is that we did not compare the serum exosome GPC1 value to immunohistochemical GPC1 expression. Additional clinical studies must be conducted for the use of serum GPC1 as a tumor marker. Another limitation is that we were unable to identify the downstream pathways of GPC1. One study demonstrated that GPC1 acts as a sonic hedgehog (Shh) co-receptor in commissural neurons [29–31]. Another study showed that GPC1 regulates hedgehog signaling in cholangiocyte in biliary atresia [29–31]. Results of some studies suggest that GPC1 acts as a negative regulator of hedgehog signaling. In pancreatic cancer, although still controversial [32–34], both clinical and experimentally obtained results of studies suggest that Shh signaling is a tumor suppressor [35–36]. Therefore, GPC1 might exert its oncogenic role by interacting with hedgehog pathway in pancreatic cancer.

In conclusion, this study demonstrated the following facts: GPC1 is expressed from precancerous lesions to invasive ductal carcinoma of the pancreas. GPC1 can functionally play an important oncogenic role. Moreover, it is regulated by EVI1 in pancreatic carcinogenesis. Overexpression of GPC1 marks the full spectrum of human pancreatic cancer precursors and PDAC as a useful early marker of pancreatic neoplasms. Furthermore, inhibition of GPC1 and/or EVI1 is an important therapeutic target in pancreatic cancer. These findings are important for the elucidation of pancreatic carcinogenesis and for the development of new diagnostic and therapeutic strategies.

## MATERIALS AND METHODS

### Cases

After reviewing The University of Tokyo Hospital pathology archives for 1986–2015, we analyzed cases of 311 PDACs, 128 IPMNs, 12 MCNs, 17 neuroendocrine tumors, 5 acinar cell carcinomas, 8 solid papillary tumors, and 20 chronic pancreatitis. In addition, 23 PanINs were selected from resected specimens. Investigations were conducted in accordance with ethical standards authorized by the Research Ethics Committee of The University of Tokyo.

### Histopathological and immunohistochemical examination

For each case, tumor tissues were processed and embedded in paraffin. All tissue slides were examined as described in earlier reports [20–21]. Tissue microarrays for PDACs were constructed as described in earlier reports [20–21]. The histological diagnosis was based on the World Health Organization classification. Immunohistochemical staining in surgically resected specimens was conducted according to standard techniques for a Ventana Benchmark^®^ XT Autostainer (Roche Diagnostics, Basel, Switzerland). GPC1 expression was evaluated according to the staining pattern and intensity. The immunostaining results of GPC1 were evaluated as negative, very weakly positive, weakly positive, moderately positive, or strongly positive. The GPC1 labeling was done according to the following system: a score of 5 if more than 50% of the neoplastic cells were labeled as having strong intensity; a score of 4 if more than 50% of the neoplastic cells were labeled as having moderate intensity or 10–50% of the neoplastic cells were labeled as having strong intensity; a score of 3 if 10–50% of the neoplastic cells were labeled as having a moderate intensity; a score of 2 if more than 50% of the neoplastic cells were labeled as having weak intensity; a score of 1 if more than 50% of the neoplastic cells were labeled as having very weak intensity, or if 10–50% of these cells were labeled at a weak intensity; and a score of 0 if fewer than 10% of the neoplastic cells were labeled at any intensity, or if fewer than 50% of these cells were labeled as having very weak intensity. Normal gastrointestinal epithelium was used as a positive control for GPC1 immunostaining. A pathologist blindly and independently evaluated the specimens for immunohistochemical analyses.

### Cell lines

The human pancreatic duct epithelial (HPDE) cell line, which was immortalized by serial passage and transduction with recombinant lentiviruses carrying HPV16 E6/E7 gene, was a kind gift from Dr. Ming Tsao, Ontario Cancer Institute. One pancreatic cancer cell line BxPC-3 was purchased from American Type Culture Collection (Manassas, VA, USA). One pancreatic cancer cell line DANG was obtained from Deutsches Krebsforschungszentrum (Heidelberg, Germany). The other 10 pancreatic cancer cell lines were obtained from Cell Resource Center for Biomedical Research Institute of Development, Aging and Cancer Tohoku University. Among pancreatic cancer cell lines, nine cell lines (PANC-1, PK-1, PK-8, PK-9, PK-45H, PK-45P, KLM-1, DANG and BxPC-3) were cultured in RPMI1640 medium (Nacalai Tesque Inc., Tokyo, Japan); two cell lines (MIA PaCa-2 and Capan-1) were cultured in Dulbecco’s modified Eagle’s medium (Nacalai Tesque Inc.) supplemented with 10% FBS, penicillin (40 U/mL), and streptomycin (50 μg/mL). HPDE cell lines were cultured in keratinocyte serum-free medium with 0.2 ng/ml EGF and 30 μg/ml bovine pituitary extract (Thermo Fisher Scientific Inc., Waltham, MA, USA).

### Antibodies and reagents

Antibodies used for immunohistochemical examination were the following: GPC1 NBP1-89759 (1:100; Novus Biologicals, Littleton, CO, USA) and EVI1 C50E12 (1:1000; Cell Signaling Technology Inc., Danvers, MA, USA).

### siRNA and miRNA duplex

The siRNAs and synthetic miRNA duplex were purchased from Thermo Fisher Scientific Inc. or Santa Cruz Biotechnology Inc. (Dallas, TX, USA), and Qiagen Inc. (Hilden, Germany) (miRNA precursor). They were introduced at 20 nM using Lipofectamine RNAi MAX (Thermo Fisher Scientific Inc.). Control siRNAs were purchased from Thermo Fisher Scientific Inc. (Stealth RNAi) or Qiagen Inc. (AllStars Negative Control). The transfected cells were used for later experiments after 72 h to 168 h. Details of siRNAs are the following: siGPC1-#1 sense, GCCCUGACUAUUGCCGAAAUGUGCU, antisense, AGCACAUUUCGGCAAUAGUCAGGGC, siGPC1-#2 sense, GCGGUGAUGGCUGUCUGGAUGAUGACCU, antisense, AGGUCAUCCAGACAGACAGCCAUCACCGC, and siEVI1 sense AUUGAAGCCAGAUUCUGAAGAGGGC, antisense GCCCUCUUCAGAAUCUGGCUUCAAU.

### Plasmids

The pME18s-EVI1 plasmids, a kind gift from Dr. Mineo Kurokawa, Department of Hematology, The University of Tokyo, Japan, were introduced at 5.0 μg using Lipofectamine 3000 (Thermo Fisher Scientific Inc.).

### RNA isolation and quantitative reverse transcription RT-PCR (qRT-PCR)

For detection of mRNA and miRNA, after RNA was extracted using ISOGEN II (Nippon Gene Co. Ltd., Tokyo, Japan), it was subjected to reverse transcription using the ReverTra Ace (R) cDNA Synthesis kit (Toyobo Lifescience Co. Ltd., Osaka, Japan). The expression levels of mature miRNAs were measured using a miScript polymerase chain reaction (PCR) system (Qiagen Inc.). Then qRT-PCR was performed with Eco Real-Time PCR System (Illumina Inc., San Diego, CA, USA) with analyses using the delta–delta Ct method. Results were normalized to β-actin for mRNA detection, and to U6 snRNA for the evaluation of mature miRNA. The following primer sequences were used: human GPC1 forward, 5′-GATGGCTGTCTGGATGACCT-3′, reverse, 5′-GTAAGGGCCAGGAAGAGGAG-3′, human EVI1 forward, 5′-AGCCTCCAGTGACACCTGCCA-3′, reverse, 5′-AGGAGTGGGTCTTGCATGCTGC-3′, human KRAS forward, 5′-AGGTGCGGGAGAGAGGCCTG-3, reverse, 5′-TGCCTACGCCACCAGCTCCA -3′, and human β-Actin forward, 5′-AGAAGGAGATCACTGCCCTGGCACC-3′, reverse, 5′-CCTGCTTGCTGATCCACATCTGCTG-3′.

### Mutational analysis of *KRAS*

The mutational statuses of the *KRAS* genes were investigated, as described previously [22]. In short, tumor DNA was extracted from formalin-fixed, paraffin-embedded tissue blocks and amplified by PCR using the primer pairs toexamine exon 2 of *KRAS* specifically, because these were previously identified as mutation hot spots 12 and 13.

### Growth assay and migration assay

Cells (3 × 10^3^ cells) were seeded in 96-well plates and were transfected with siRNAs. After 0, 24, 48, 72, and 96 h, cell viability was quantified by colorimetric assay using WST-8 (Nacalai Tesque Inc.). For HPDE cell lines, cells were seeded in six-well plates with transfection of siRNA and were counted with trypan blue staining every 24 hours for 13 days. For *in vitro* wound-healing assays, cells were grown to confluence and were then damaged with a plastic pipette tip. The wound area was then photographed and photographed again every 24 h thereafter. The migration distance was found using computer-driven image analysis.

### Expression analyses

Whole gene expression pattern was determined by gene expression array (Agilent Technologies). Briefly, Cy3-labeled synthesized cRNA samples were hybridized on 4 × 44K Whole Human Genome Oligo Microarray (Agilent Technologies). The signals were detected by the microarray scanner (Agilent Technologies G2565BA) and analyzed by GeneSpring 12.5 (Agilent Technologies). Prior to comparative analyses in gene expression profiles among tested samples, we conducted appropriate normalization on the set of raw data, i.e., (1) data transformation: set measurement less than 0.01 to 0.01; (2) per chip: normalized to 50th percentile; and (3) per gene: normalize to mean. mRNA expressed at significantly different levels among each group were identified by filtering on fold-change. Correlations between two groups were analyzed by Pearson’s correlation coefficients. Gene ontology analysis was performed by DAVID (http://david.abcc.ncifcrf.gov/).

### Statistical analysis

Statistical analysis was performed using chi-square test and Fisher’s exact test. *p* < .05 was regarded as statistically significant (^*^p < .05). Survival curves were constructed using the Kaplan–Meier method with software (JMP Pro 11.2.0; SAS Institute Inc., Cary, NC, USA).

## SUPPLEMENTARY MATERIALS FIGURE



## Abbreviations

EVI1: ecotropic viral integration site 1
GPC1: glypican-1
HPDE cell: human pancreatic duct epithelial cell
IPMN: intraductal papillary-mucinous neoplasm
MCN: mucinous cystic neoplasm
miRNA: microRNA
PanIN: pancreatic intraepithelial neoplasia
PDAC: pancreatic ductal adenocarcinoma
