# Supplementary material for: EVI1 modulates oncogenic role of GPC1 in pancreatic carcinogenesis
Source: Oncotarget. 2017 Sep 1;8(59):99552–66. doi: 10.18632/oncotarget.20601 (PMC5725114; doi:10.18632/oncotarget.20601)
Supplement: Supplementary file 1 [file oncotarget-08-99552-s001.pdf]

EVI1 modulates oncogenic role of GPC1 in pancreatic carcinogenesis

SUPPLEMENTARY MATERIALS

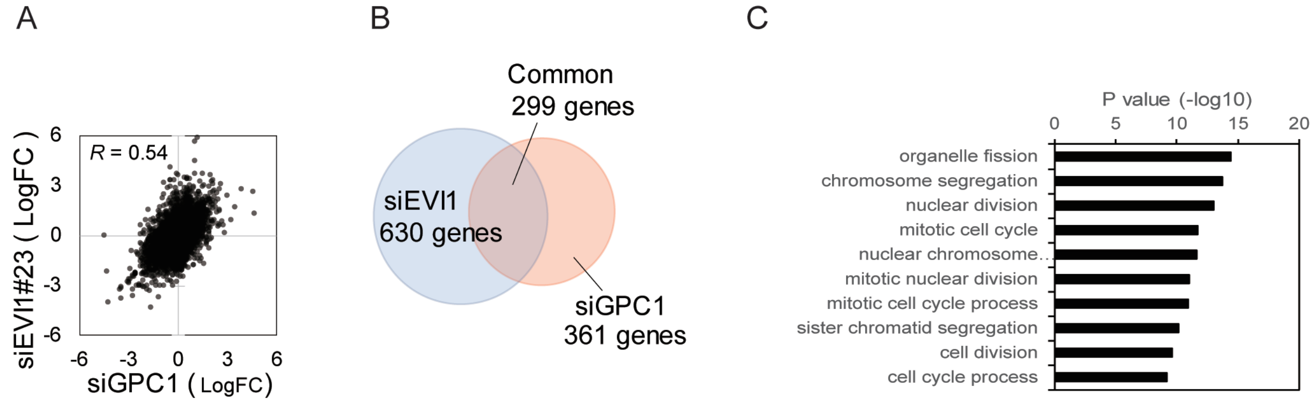

Supplementary Figure 1: (**A**) Scatter plot showing all gene expression changes (LogFC) by siGPC1 (x-axis) and siEVI1 (y-axis) in PK-45H cell lines. (**B**) Venn diagram showing genes significantly downregulated in siGPC1 group (red circle, FC<-2, 660 genes) and siEVI1 group (blue circle, FC<-2, 929 genes). (**C**) Gene ontology analysis of common 299 genes which were downregulation both in siGPC1 group and siEVI1 group in PK-45H cell lines. Significant GO terms and their P values are shown by a bar graph.
